# A Normative Feedback Intervention on Gambling Behavior—A Longitudinal Study of Post-Intervention Gambling Practices in At-Risk Gamblers

**DOI:** 10.3389/fpsyt.2022.602846

**Published:** 2022-03-31

**Authors:** Jonas Berge, Tove Abrahamsson, Axel Lyckberg, Katja Franklin, Anders Håkansson

**Affiliations:** ^1^Department of Clinical Sciences Lund, Psychiatry, Faculty of Medicine, Lund University, Lund, Sweden; ^2^Malmö Addiction Center, Malmö, Sweden; ^3^Division of Primary Care, Malmö, Sweden; ^4^AB Svenska Spel, Göteborg, Sweden

**Keywords:** gambling disorder, online gambling, problem gambling, normative feedback, motivational intervention, behavioral feedback

## Abstract

**Background:**

In problem gambling, normative personalized feedback interventions have demonstrated promising effects. Given the widespread increase in online gambling in recent years, internet-delivered normative feedback may serve as a promising intervention. This study aimed to examine whether such an intervention, delivered by a gambling operator and aiming to help problem gamblers decrease their gambling, may in fact be associated with lower gambling practices post-intervention.

**Methods:**

Online questions on norms and beliefs about one's own and peers' gambling habits, derived from the Gambling Quantity and Perceived Norms Scale, were followed by personalized feedback, delivered online by the Swedish state-owned gambling operator. A total of 1,453 gamblers consented to participate in a pre-post measure of wagering levels.

**Results:**

Wagering decreased significantly post-intervention (28 days) compared to pre-intervention (28 days prior). The decrease was significantly more pronounced in younger and online casino gamblers. In an 84-day follow-up, the decrease remained significant, although less pronounced.

**Conclusions:**

An online normative intervention delivered by a state-owned gambling operator, addressing norms and beliefs about gambling levels, may lower risky gambling in the short term. Implications and further research needs are discussed.

## Background

Problem gambling, including the diagnostic construct of a gambling disorder (1), affects a significant minority of the population worldwide, with prevalence estimations ranging up to ~5% of the population (2, 3). A gambling disorder diagnosis is typically characterized by a gambling pattern involving increasing amounts of money, a “chasing losses” behavior (i.e., where a person returns to gambling primarily in order to try to win back the money lost), lying to family members and friends, and continued gambling despite negative consequences (1). Gambling disorder may be associated with severe social and health consequences (4), including comorbid psychiatric diagnoses, psychological distress as a consequence of debts (5, 6), suicidal ideation (7), and an increased risk of suicide (8). Despite increasing scientific support for treatment involving cognitive–behavioral therapeutic approaches, treatment seeking for problem gambling has been described to be low (9, 10). In most settings, a majority of problem gamblers are men (2), although, in recent years, scholars have argued that high-risk gambling has become more acceptable among women and that the difference in the prevalence of gambling problems between men and women may be decreasing (6, 11–14).

In recent years, researchers have increasingly highlighted the role of gambling operators in primary and secondary prevention of problem gambling, through different responsible gambling measures. These may include interventions addressing problem gambling in close proximity to the gambling situation, such as through direct communication from a gambling operator detecting a pattern of problem gambling (15–17). One opportunity for brief intervention in problem gambling may be to address individuals' beliefs about their gambling in comparison to the gambling patterns of their peers. It has been suggested that, in the general population, when assessing beliefs about the extent of peers' gambling, problem gamblers report beliefs about more intense gambling in their peers than do non-problem gamblers (18). Studies have also shown that many college students tend to overestimate the gambling expenditure of their peers, and also that these overestimations are positively associated with the students' own frequency of gambling, their gambling expenditures, and gambling-related harm. Thus, discrepancies between perceived and actual norms for college gambling are of relevance to college students' gambling behaviors and gambling-related problems (19, 20). Personalized feedback interventions, addressing gamblers' beliefs about their own gambling compared to the gambling of their peers, have been reported to have promising effects on measures of problem gambling (21, 22).

The theory behind the intervention relies on the assumption that individuals may experience misperceptions about how much other people—in life situations similar to their own—gamble. This theory has previously been applied in the field of alcohol use disorders and has expanded to the field of problem gambling. The rationale of a personalized, normative feedback intervention in gambling is theoretically to help individuals reflect on their own levels of gambling, possibly in order to correct such misperceptions and in order to help them decrease their gambling (21).

Previous studies addressing normative interventions in gambling have included mainly university students or general young adult populations. Few studies have assessed these interventions among customers of a gambling operator (22–25). In a study on online interventions in poker gamblers, a brief personalized normative feedback was limited by high dropout in the study but showed acceptability comparable to more elaborate therapeutic interventions (26). In another study, Auer and Griffiths provided promising findings from a voluntary behavioral feedback intervention system at a gambling operator's site (15). Theoretically, when provided directly by a gambling operator, interventions aiming to help at-risk gamblers reduce or discontinue their gambling can be provided in closer temporal association with a gambling session than could any other motivational or therapeutic intervention, such as those provided by a service offering treatment or support. The opportunity for interventions in close association with the gambling situation is a potentially important part of the responsible gambling strategies of gambling operators. The concept of providing gambling, while maintaining primary and secondary preventive tools for gambling problems, has been described in a limited number of publications. Examples of such interventions include direct feedback and motivational contact from state-owned gambling operators to clients presenting a potentially hazardous gambling behavior (17) and gambling-reducing measures such as loss limits (27).

The present study aimed to address gamblers' norms and beliefs about their own gambling habits and those of their peers and thereby intended to assess whether interventional feedback on these beliefs has the potential to decrease gambling when delivered by a gambling operator as a responsible gambling intervention. This approach could potentially reach at-risk gamblers in direct association with their gambling and independent of treatment settings. The study intervention, consisting of a normative test intended to stimulate gamblers' own reflections and motivational processes, was delivered to clients of the state-owned Swedish gambling operator AB Svenska Spel, either because the clients were shown to be at risk of problem gambling or because they actively sought this kind of normative testing. More specifically, the study aimed to assess whether a normative test and the delivery of feedback may lower the level of wagering and which factors, such as gender, age, type of gambling, and the reason for taking the test, would be associated with decreased wagering post-intervention. The primary aim of the study was to address changes in wagering during a post-intervention period corresponding to the time frame studied pre-intervention (4 weeks). Additionally, this study examined whether a potential decrease in wagering may persist during 12 weeks after the intervention.

## Materials and Methods

### Study Design

This was a longitudinal study measuring gambling patterns prior to and after an online-based intervention to clients of the state-owned gambling operator AB Svenska Spel. The study was conducted in a collaboration between Lund University and AB Svenska Spel. The present study was carried out in the subsection of AB Svenska Spel providing gambling on various types of sports betting, as well as an online casino, bingo, and poker (*Svenska Spel Sport & Casino*). The rationale for the analyses in these forms of gambling is the suggested high addictive potential of sports betting and online casino gambling in treatment-seeking patients in the present setting (28).

### Setting

Following a new regulation in use since January 2019, the Swedish gambling market is a licensed market. Gambling operators are granted licenses from a governmental authority, provided they follow a number of universal responsible gambling policies including the adherence to a nationwide self-exclusion system, where a person can self-exclude from all licensed gambling operators in Sweden (29). AB Svenska Spel is the only gambling operator owned entirely by the state and operating under instructions from the Swedish government. In Sweden, online gambling has increased steeply during the past decade and represents the most common gambling modality in television advertisements (30) and the most commonly reported by treatment-seeking problem gamblers (28). Problem gambling has recently been reported to increase in the Swedish population, with the most pronounced increase seen among women (31). Gambling habits, as well as gambling problems, are known to differ substantially between women and men (6, 11–14). Likewise, it has been reported that the characteristics of problem gambling differ by age; problem gambling in the present setting is more common in the young (13), and personality factors, psychiatric comorbidity, and the overall clinical picture in problem gambling have been described to differ by age (32, 33).

The present study is based on a normative test provided as part of the responsible gambling tool *Playscan*, used by the state-owned Swedish gambling operator AB Svenska Spel. The *Playscan* tool has previously been described in scientific papers (34, 35). *Playscan* was a sub-department of AB Svenska Spel at the time of the study and a brand name describing a behavioral tracking tool that provides a weekly individual risk assessment, advice, and strategies on how to keep track of gambling behavior. The *Playscan* user interface holds several self-tests, related to responsible gambling, where users can investigate and reflect upon their gambling habits. The tool is accessible to the user on AB Svenska Spel's website and uses an on-site notification system to get the users' attention. The *Playscan* tool has been operating since 2007 and is fully owned by AB Svenska Spel and also has been applied by other gambling operators internationally during the past decade. In 2010, the French gambling operator La Française des Jeux added *Playscan*. A year later, it started to be used by the Swedish lottery Miljonlotteriet. In 2014, the state-owned Norwegian operator Norsk Tipping started to use *Playscan*. In 2019, Loterie Romande in Switzerland launched the tool to all its players.

### Intervention

The present study assessed the change in gambling behavior following a normative feedback intervention that was a part of the preexisting responsible gambling tool *Playscan*. The normative test was taken at any time during the period January 28 through April 8, 2019. Players could enter the normative test through any of two pathways: (1) an on-site notification offered to players who had gambled with a theoretical loss (36) of at least 500 Swedish kronor (SEK, corresponding to ~45 Euros) during the past 5 weeks and with no previous activity in *Playscan* (passive method) or (2) by actively clicking on any of several links to the test inside the *Playscan* user interface (active method). The former group included players with possible high-risk gambling behavior identified by *Playscan*'s weekly risk analysis. A gambling pattern associated with risk, according to *Playscan*, was defined as an escalation of time and/or money spent on gambling over time (35). In the test, the client was asked to report the gambling type that she/he wished to be compared for (sports bettor, bingo gambler, online casino gambler, poker gambler, or various) and her/his level of gambling experience (beginner, average, or advanced). Thereafter, the test consisted of the following questions derived from the Gambling Quantity and Perceived Norms Scale [GQPN, (37)]: the client's frequency of gambling (days per month), her/his gambling losses during a typical month, beliefs about peers' frequency of gambling and typical monthly loss for the same gambling type (ranging from < SEK 50 to >SEK 50,000), and the client's estimated loss during the past month. When the client had answered all the questions, feedback was presented in the form of a summary of her/his responses, which were then compared to actual data on the frequency of playing and average monthly loss for a typical player of the gambling operator's *Sports and Casino* sub-division.

### Ethical Considerations

Data on wagers, winnings, and losses for all players who use the gambling web page are registered and stored by AB Svenska Spel. All players who entered the test were asked for consent to include this data, for the past 90 days and the following 90 days in relation to the consent, as well as data from the test, in the study. Players who declined to give consent could still take the full test, outside of the study. Entirely anonymized data were delivered to co-authors JB and AH for statistical analyses. The study was reviewed and approved by the regional ethics board, Lund, Sweden (file number 2018/699).

### Study Periods

For each study participant, the amount of money wagered and the net win or loss were logged for 90 days prior to and 90 days following the intervention. However, data registration started on January 1, 2019, and the study started on January 28, 2019. This means that all study participants who were included earlier than the 90th day of 2019 (March 31) would have missing values for any gambling taking place before 2019. We therefore chose not to use any of the data prior to 28 days before the study started for each individual. Furthermore, due to the nature of the brief intervention and the hypothesized short duration of any potential effect, we limited the follow-up time to 28 days following the intervention. We used the amount of wager as the basis for our analyses because unlike net win or loss, which has negative values, a wager can be logarithmized. We thus used data on wager during the 28 days prior to and the 28 days following the intervention for each study participant. In a secondary analysis, the same pre-intervention period was applied (for the reasons stated above), whereas the post-intervention period studied included 12 weeks (84 days) post-intervention.

### Outcome Variable: Average Daily Wager

The outcome measure used in the study was a change in wager following the intervention, i.e., the total amount of money wagered per day for each study participant (in SEK). Because of the large proportion of days with no money spent on gambling (a total of 59.0% of all days for all study participants), the data had to be reduced in order to avoid an excessive number of zeroes in the model. We thus averaged the 56 days of observation for each participant into eight 7-day periods, four before the intervention (periods 1–4) and four after the intervention (periods 5–8), and calculated the average amount of money wagered per day within each period [average daily wager (ADW)]. In total, in only 18.8% of such periods did a study participant not spend any money on gambling.

The study participants might have started gambling at AB Svenska Spel's websites at any point prior to the intervention. We included only 97.1% of study participants who had at least one gambling occasion during the 28 days prior to the intervention (*n* = 1,411). We had no data on whether the clients' first gambling occasion during the study period was their first-ever gambling occasion at the *Sports and Casino* website. Eighty percent of this group of participants had their entry into the study (i.e., the first gambling occasion during the study period) in the first period and 10% in the second period. Because of the different pathways to inclusion in the study outlined above, there was a potential need to control for the effect of the pathway on the estimated association between the intervention and gambling behavior. More specifically, individuals who lost high amounts of money in a single session might have been included in the study immediately after the loss, and the subsequent absence of gambling might not have been related to the intervention but instead, for example, to a lack of money to wager. For this reason, we excluded all participants who had their first gambling occasion in the fourth period (*n* = 66). In order to handle potential outliers (i.e., study participants who wagered extremely high amounts of money compared to the median), we excluded all participants who were in the top 1% of the wagered amount in any of the eight periods. This corresponded to 3.9% (*n* = 53) of the individuals remaining after having been excluded for the reasons outlined above. The final number of study participants was thus 1,292, corresponding to 88.9% of the individuals who provided informed consent for participation in the study.

In order to model differences based on the period in which study participants had their first gambling occasion, we used a variable describing the first gambling period as a covariate in the regression model.

Finally, the ADW values were logarithmized in order to approach a normal distribution more suitable for use in a multiple regression model. Because the logarithm of 0 is negative infinity, a value of SEK 1 was added to all periods for all participants, with the exception of periods prior to the first period of gambling for each individual, which were excluded from the analyses.

All ADW values were calculated and reported in local currency (Swedish krona, SEK). For improved clarification of the magnitude of findings, values were translated into US dollars (USD), where nine SEK correspond to 1 USD.

### Covariates

The covariates used in the present study were the following:

- Gender: female, male.
- Age at the intervention: we used data on birth year to calculate the approximate age for each participant based on the fact that all interventions took place during <3 months, so the errors should be minimal. Age was divided by 10 and centered at the median, 39, so that the estimated value in the regression model reflects the effect of each additional 10 years higher than the median.
- Method of entry into the study: method of entry was dichotomized into passive and active methods, with passive indicating a notification from the gambling site and active requiring that the participant sought out the intervention actively. The passive method was used as the reference because it was far more common.
- Intervention feedback: as described above, participants who completed all the questions in the intervention received feedback on their accuracy when estimating how much other people spend on gaming. We dichotomized the completers into two groups; those who estimated somewhat correct with respect to others' gambling behavior (estimation less than twice the actual value) and those who highly overestimated others' gambling behavior (twice or more than the actual value). A tripartite intervention feedback variable was thus created, with non-completers, average estimators, and overestimators representing the different categories.
- Self-assessed main type of gambling interest: the alternatives that players could indicate as their main type of gambling were sports betting, bingo, online casino, poker, and others. For purposes of the study, *sports betting* was defined as the reference because it was by far the largest category, and *casino* (the second largest category) and *other* (including all other types) were defined as dummy variables in order to be compared to sports betting.

### Statistical Methods

In order to assess the change in ADW following the intervention, we first created a regression model that estimated the global association, without including any of the covariates of interest. This was a mixed model multiple regression model, with a random intercept for each individual, and will henceforth be referred to as the *structural submodel*.

**Figure 1:**
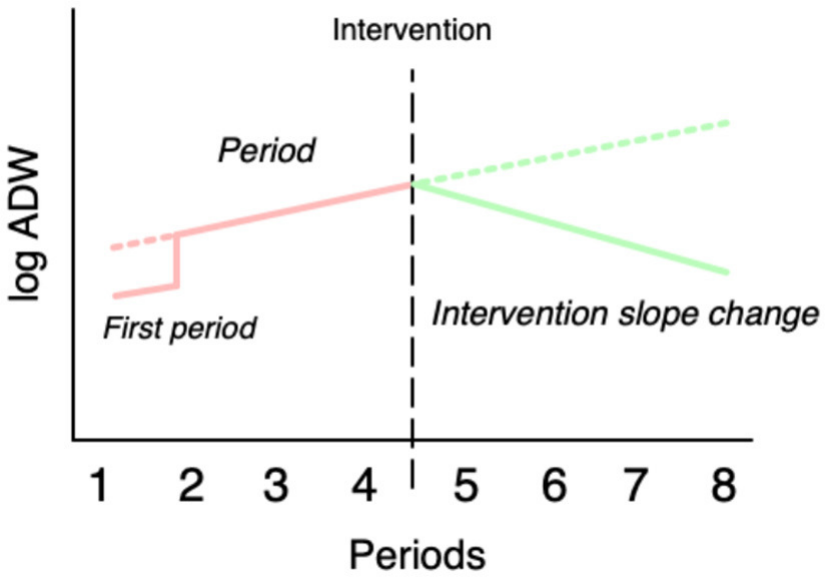
Schematic illustration of the variables used in the main regression mode.

The first set of analyses included a follow-up period of 4 weeks, corresponding to a pre-intervention period of the same duration. As described above, each individual had six to eight periods of measurement. The log ADW for each period was used as the dependent variable. The first independent variable in the structural submodel was *period*, ranging from 1 to 8, with 1–4 referring to the periods prior to the intervention and 5–8 to the periods following the intervention. The second independent variable, first *period*, was a categorical variable defined as the period in which each individual in the study population had his/her first gambling period in the data set, as described above. We devised two strategies to model the change associated with the intervention. The first strategy, *intervention mean change*, was to estimate the shift of the intercept with a variable defined as 0 for all periods pre-intervention and 1 for all periods post-intervention. The second strategy, *intervention slope change*, was to estimate the shift of the slope of the curve following the intervention, and this variable was defined as 0 for all periods pre-intervention and 1–4 for the periods post-intervention. We then ran models with all combinations of *period*, first *period, intervention mean change*, and *intervention slope change* including at least one of the intervention variables and compared the resulting 12 models on the Akaike information criterion and Bayesian information criterion. Both methods favored the model with *period*, first *period*, and *intervention slope change* (Supplementary Table S1). The structural submodel can thus be expressed as in Figure 1:

*log (ADW)* = *intercept* + *period* + *first period* + *intervention slope change* + *random intercept*

All covariates were added to the structural submodel both as an estimate of the effect of the variable itself and as an interaction effect between the intervention and the variable in question, in order to assess whether the intervention had different effects across different levels of each variable. The full adjusted model thus included a total of 17 fixed variables (5 for the structural submodel, including the intercept, 7 for the covariates, and 7 for the interaction terms) and one random intercept.

In a secondary analysis, the same methodology as above was applied, although with a follow-up period of 12 weeks (84 days) after the intervention, instead of 4 weeks. As previously, the pre-intervention period included 4 weeks.

Three sensitivity analyses were performed on the full model involving the 4-week follow-up. In the first sensitivity analysis, individuals with a first gambling period other than 1 (15.9%, n = 206) were excluded from the analysis. In the second sensitivity analysis, all periods with an ADW of 0 were excluded (13.7%, 1,211 of 8,851 periods), and in the third sensitivity analysis, SEK 10 was added to ADW instead of SEK 1 as in the full model. In none of the sensitivity analyses was the estimate of the main intervention effect altered substantially, and neither were the interaction effects between intervention and age and between intervention and overestimation of others' gambling. In the second sensitivity analysis, the interaction effect between the intervention and preference for the online casino was diminished (from −0.15 to −0.06) and no longer statistically significant. The interaction effect between the intervention and female gender was diminished in all the three sensitivity analyses and no longer statistically significant (Supplementary Table S2).

All data management and analyses were performed in R 3.5.3 (38).

## Results

A total of 3,432 individuals entered the test and responded to the question about consent to include their data in the study. Of these, 1,453 individuals consented, after applying the exclusion criteria described in the *Methods* section. Among these, 84.1% had their first gambling day within the first period (22–28 days prior to the intervention), 10.7% within the second period (15–21 days prior to the intervention), and the remaining 5.3% within the third period (8–15 days prior to the intervention). Participants were predominantly male, with only 6.4% women. The median age was 39 years, ranging from 18 to 90 years with an interquartile range of 30–51 years. The most common method of entry into the study was by automated notification by the website (passive), accounting for 73.6% (*n* = 951) of the participants and 25.5% (n = 331) actively clicked on a *Playscan* link. A total of 11 participants had a different method of entry (notification by risk profiling) and were excluded from the main analysis.

**Figure 2:**
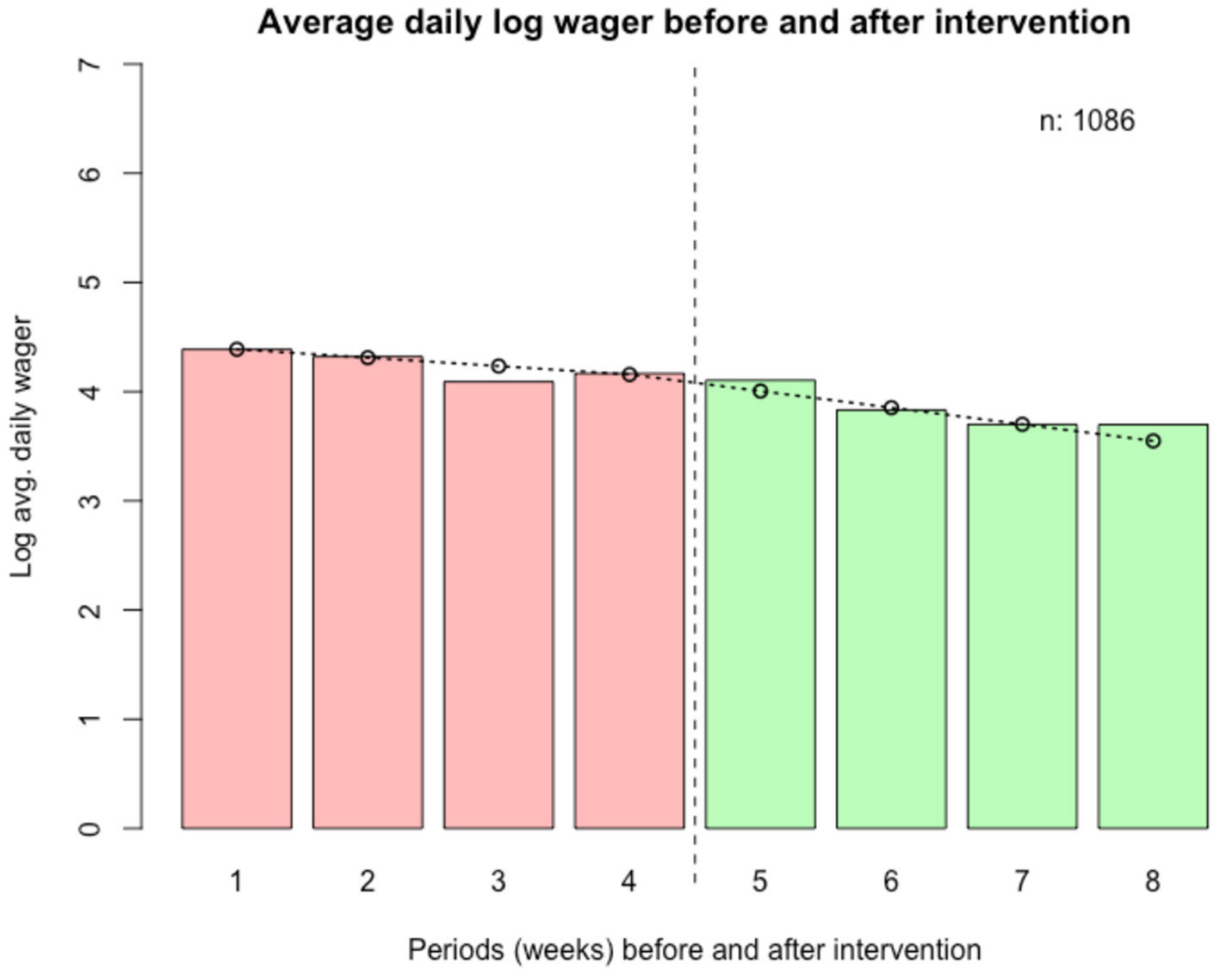
Wager data pre- and post-intervention, logarithmized. Weeks before (weeks 1–4) and after (weeks 5–8) intervention.

The median ADW was SEK 74.1 (~USD 8.2) in the 28 days prior to the intervention and SEK 57.7 (22% lower, ~USD 6.4) in the 28 days following the intervention. The logarithmized ADW for the periods before and after the intervention is shown in the histograms in Figure 2. As can be seen in the figure, there is a spike of very low values (because SEK 1 was added to all values of ADW) in the periods prior to the intervention and a larger spike of zeroes in the periods following the intervention. The second and third sensitivity analyses described in the *Methods* section were performed for this reason.

**Figure 3:**
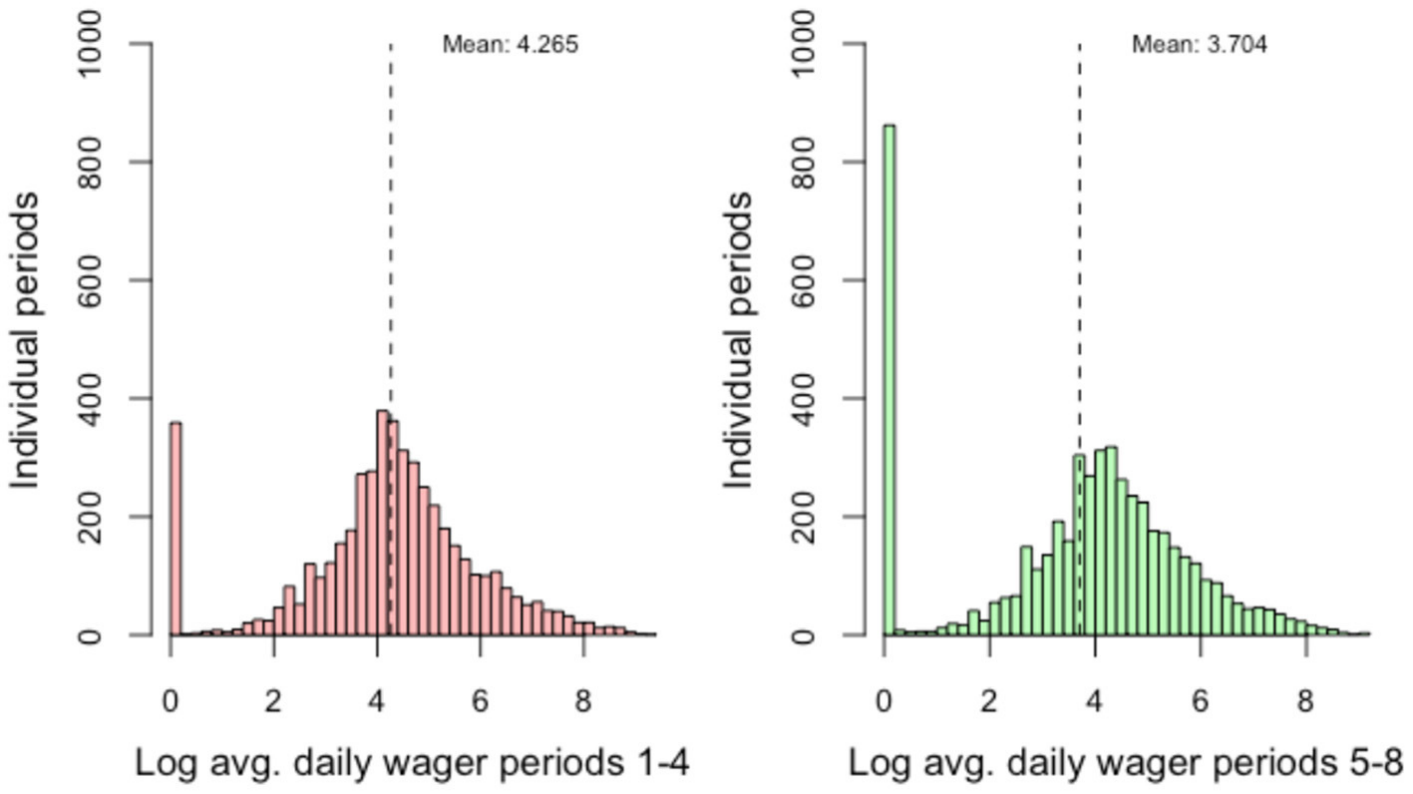
Average daily log wager before (28 days) and after (28 days) intervention.

In Figure 3, the mean logarithmized ADW is shown for all periods before and after the intervention, as well as a line representing the predicted values from the main regression model.

The results from the mixed model multiple regression models are shown in Table 1. In the second column, the results from the structural submodel are shown. The conditional R squared for this model was 0.382, and the marginal R squared was 0.033. In this model, there was a clear statistical effect of period, first period, and the intervention on the log ADW. The absolute statistical effect of the intervention was about equal in size to the effect of the period variable. In the full model, in which all covariates and interaction terms between the covariates and the intervention were included, the statistical effect of the intervention was considerably larger than the effect of period (−0.29 vs. −0.08). The conditional R squared for this model was 0.385, and the marginal R squared was 0.050.

**Table 1:** Mixed model regression models on log ADW as dependent variable.

**Variables**	**Structural submodel**	**Full model**	
	**Estimate (95% CI)**	**Estimate (95% CI)**	***p*-value**
Period	−0.07 (−0.11, −0.04)	−0.08 (−0.11, −0.04)	<0.001
First period			
1 (reference)		*1*	
2	−0.44 (−0.67, −0.21)	−0.32 (−0.55, −0.09)	0.006
3	−0.72 (−1.04, −0.40)	−0.58 (−0.91, −0.26)	<0.001
Intervention slope change	−0.09 (−0.14, −0.03)	−0.29 (−0.39, −0.19)	<0.001
Female sex		−0.08 (−0.39, 0.23)	0.610
Age (per 10 years)		0.04 (−0.02, 0.09)	0.163
Entry method		−0.27 (−0.44, −0.09)	0.002
Preferred gambling type			
Betting (reference)		1	
Online casino		0.41 (0.01, 0.81)	0.046
Other		−0.12 (−0.33, 0.08)	0.237
Intervention feedback			
Moderate estimation		1	
Over-estimation		0.32 (0.10, 0.54)	0.004
Non-completer		0.15 (−0.07, 0.37)	0.187
Intervention x Sex		−0.10 (−0.19, −0.02)	0.021
Intervention x Age		0.05 (0.04, 0.07)	<0.001
Intervention x Entry		0.00 (−0.04, 0.05)	0.895
Intervention x Casino		−0.15 (−0.26, −0.04)	0.007
Intervention x Other		0.04 (−0.02, 0.09)	0.169
Intervention x Over-estimation		−0.05 (−0.11, 0.01)	0.120
Intervention x Non-completer		−0.02 (−0.08, 0.04)	0.457

*Four-week follow-up post-intervention*.

There was no linear association between log ADW and gender or age. However, there was a statistically significant interaction effect between the intervention and age; this was associated with an increase of the log ADW following the intervention with 0.05 per period (95% CI: 0.04, 0.07) for every 10 years of age in addition to the median age of 39, and, consequently, with −0.05 for every 10 years of age below the median age. Likewise, the interaction effect between the intervention and female gender was significant, with a decrease of log ADW of −0.10 (95% CI: −0.19, −0.02). Participants with casino as the preferred gambling type, compared to participants with sports betting as the preferred type, had a higher log ADW as well as a steeper decrease of log ADW at −0.15 (95% CI: −0.26, −0.04) per period following the intervention (interaction effect). There were no statistically significant differences between participants with “other” as the preferred gambling type when compared to those who reported sports betting as the preferred type.

Study participants who overestimated how much others spend on gambling had a higher log ADW than those who made more moderate estimations (0.32, 95% CI: 0.10, 0.54), but neither overestimators nor non-completers had a significantly different effect of the intervention than the reference group.

In a *post-hoc* analysis, we created five equally sized groups based on ADW for the 28 days prior to the intervention (i.e., percentiles <20, 20–40, 40–60, 60–80, and 80 and above). The median ADW in SEK for the groups was 35, 63, 98, 185, and 616. The median change following the intervention in SEK for each of the groups was −1 (3%), −9 (14%), −19 (19%), −56 (30%), and −233 (38%). When assessing the mean values, the result for the highest group is striking. In that group, the mean ADW prior to the intervention was SEK 922, and the mean change in ADW following the intervention was SEK 305 (i.e., a 33% decrease). This corresponds to a wagered amount of SEK 25,825 during the 4 weeks prior to the intervention and SEK 17,293 during the 4 weeks following the intervention.

**Figure 4:**
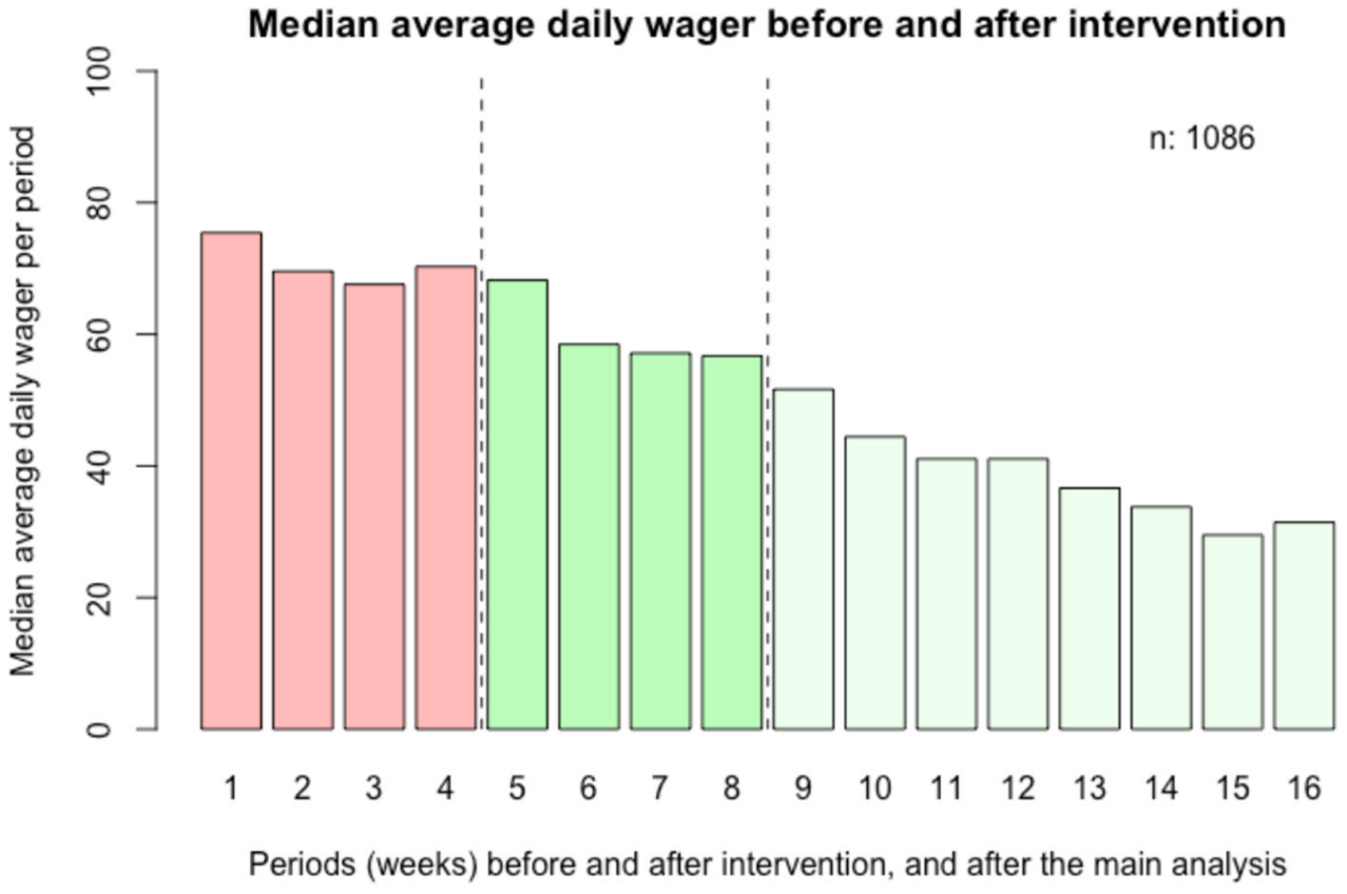
Secondary analysis; average daily log wager before (28 days) and after (84 days) intervention. Weeks before (weeks 1–4) and after (weeks 5–16) the intervention.

Results from the 12-week follow-up are demonstrated in Table 2 and Figure 4. Here, the overall decrease in ADW (intervention slope) remained significant (*p* < 0.001), but with a less steep slope (−0.11, 95% CI: −0.15, −0.07) than in the 4-week follow-up. In this model, online casino gambling was no longer significantly associated with decreased ADW. Also, the interaction of intervention slope and gender was no longer significant.

**Table 2:** Mixed model regression models on log ADW as dependent variable.

**Variables**	**Full model**	
	**Estimate (95% CI)**	***p*-value**
Period	−0.11 (−0.14, −0.08)	<0.001
First period		
1 (reference)	*1*	
2	−0.63 (−0.88, −0.39)	<0.001
3	−0.62 (−0.96, −0.28)	<0.001
Intervention slope change	−0.11 (−0.15, −0.07)	<0.001
Female sex	−0.19 (−0.52, 0.14)	0.259
Age (per 10 years)	0.06 (0.00, 0.12)	0.042
Entry method	−0.25 (−0.43, −0.07)	0.007
Preferred gambling type		
Betting (reference)	1	
Online casino	0.29 (−0.14, 0.71)	0.185
Other	−0.10 (−0.32, 0.11)	0.342
Intervention feedback		
Moderate estimation	1	
Over-estimation	0.26 (0.03, 0.49)	0.027
Non-completer	0.10 (−0.14, 0.33)	0.422
Intervention x Sex	0.00 (−0.02, 0.02)	0.894
Intervention x Age	0.02 (0.02, 0.03)	<0.001
Intervention x Entry	0.00 (−0.01, 0.01)	0.711
Intervention x Casino	−0.05 (−0.08, −0.02)	0.001
Intervention x Other	0.01 (−0.01, 0.01)	0.242
Intervention x Over-estimation	0.01 (−0.01, 0.02)	0.523
Intervention x Non-completer	0.00 (−0.02, 0.01)	0.662

*Twelve-week follow-up post-intervention*.

## Discussion

The present study demonstrated an association between an online intervention addressing norms and beliefs about gambling in individuals with a potentially hazardous gambling pattern, and a subsequent change in gambling behavior. The association between the intervention and the subsequent reduction in wagering, over 4 weeks post-intervention, was stronger in younger individuals and in online casino gamblers and lower in non-casino gamblers. However, while these interactions were statistically significant, they were diminutive when compared to the magnitude of the overall association between the intervention and the outcome measure. The sensitivity analyses, designed to assess the robustness of the results under various alternative conditions, did not alter this main finding substantially, which lends support to the validity of the association. In the longer analysis of 12 weeks post-intervention, the reduction in wagering remained significant but less pronounced than in the 4-week model, and a significant association between decreased wagering and online casino gambling was no longer seen.

In the present setting, online casino gambling has been shown to play a particular role in problem gambling in recent years. Online casino is the most common type of gambling reported by treatment seekers in a clinical setting (28), and in a recent survey, in a sample of online gamblers, recent online casino gamblers were considerably more likely to fulfill criteria for problem gambling, compared to online gamblers reporting other typologies of gambling (6). Thus, an online casino may have a closer link to problem gambling than other types and modalities of gambling, at least in a setting where gambling is predominantly carried out online. The features of online gambling are known to be particularly addictive; return-to-player rates are high, and time between wagered money and the result, and to the next wagering, is minimal. Thus, loss of control may be particularly pronounced in this type of gambling. It remains to be understood why this type of gambling, where the level of wagering was higher, was associated with a larger decrease in wagering during the first weeks after the intervention, while it did not remain a significant predictor of decreased wagering later during the follow-up.

In the present study, gender was not significantly associated with wagering, and while there was a small interaction between the intervention and female gender, this association was diminished and not statistically significant in the sensitivity analyses, casting doubts on the validity of this result. Gender is a factor known to influence gambling and problem gambling to a large extent; women and men tend to gamble on different types of games and modalities (39), but also have different courses in the development of problem gambling, with a later onset (40) and higher psychiatric comorbidity in women (28, 41, 42). While problem gambling traditionally is more common in men than in women, some previous data suggest the opposite trend in the present setting when only online gamblers are studied (6). Based on the present findings, a normative intervention in online gambling appears to be promising for both genders, when controlling for the type of gambling reported and possibly associated with a slightly larger reduction in wagering in women.

Likewise, the reduction in wagering post-intervention was larger among younger individuals. Few comparable interventions, although not delivered in the same context as here, have been tested in studies where age has been used as a co-factor to control for (15), where findings have been conflicting (16), or where age has been aimed to be similar across intervention and control groups (17, 27). Thus, the present study finding of a larger reduction in wagering among younger individuals, following a normative feedback intervention, needs to be replicated in future studies. Problem gambling is common in the young, as shown in general population data from the present setting (43), and has been shown to be associated with poorer life satisfaction (44) and with other psycho-pathological features (45). This clearly underlines the importance of further intervention research addressing young gamblers, and where mental health and life satisfaction also can be addressed.

The mode of entry into the study was of no significant importance to the change in wagering. Thus, the change in gambling behavior following the studied intervention did not differ depending on whether the gambler herself/himself sought the risk assessment, which constituted the way into this intervention, or whether the intervention was initiated by the gambling operator identifying the potentially risky gambling behavior. This finding supports the possible use of the present intervention in both conditions. Also, the promising associations seen here, regardless of how the gambler was introduced to the intervention, lend support to the use of responsible gambling practices overall by a gambling operator. This may be particularly important given the low treatment seeking in gambling disorder (10), which leads to the rationale of using the gambling situation as a window of opportunity for motivational or supporting messages to the gambler.

An important aspect of the results is the degree of the clinical utility of the decreases in wagering demonstrated. The figures presented (SEK 74.1 and 57.7 before and after the intervention, respectively) are median values in a highly skewed distribution. The mean values pre- and post-intervention were SEK 261 (~USD 29) vs. SEK 208 (~USD 23), which can be translated to a monthly difference of about SEK 1,490 (~USD 166). The magnitude of the decrease in wagering seen in the study should be seen in the context of the intervention being brief and automated and the short-term follow-up in the study. Thus, given the relatively limited intervention delivered here, it can be argued that a decrease of SEK 1,490 (~USD 166) in wagering is relatively substantial. More studies are needed using this type of normative feedback intervention delivered by a gambling operator, but the decrease seen in association with the intervention here can be interpreted at least as promising. While the results should be interpreted with caution, the decrease seen in association with the intervention here can be interpreted at least as promising. More studies are needed, in other settings and with longer follow-up periods, using this type of normative feedback intervention delivered by a gambling operator.

Implications of the present study include the strengthened support for the feasibility of addressing gamblers' attitudes and beliefs about gambling in future interventions aiming to reduce at-risk gambling. Based on previous findings, the present study aimed to test the promising normative intervention model also when provided by the gambling operator itself. While this was found in this nonrandomized controlled study design, although with non-completers as a control condition, this proved to be at least promising for further study and further use by gambling operators as part of their responsible gambling strategies. In particular, the present findings may have implications for gambling operators in other settings with a high level of online gambling, i.e., where the intervention can be delivered—and measured—within the framework of online interaction. Although beyond the scope of the present study, it also supports further studies on normative feedback interventions particularly in online casino gamblers in treatment and support settings. It is known that individuals with problem gambling may seek treatment or help in many different ways and that this type of interactive online contact may represent one of these strategies (46).

The present study translates previously promising findings in normative feedback interventions to the setting of a gambling operator as part of its responsible gambling practices in their relationship with its customers, in contrast to previous studies addressing either university students or adults from the general population screening positive for problem gambling (22). Also, this study translates these previous findings into the present setting where gambling is online in many cases, and where the online setting provides an opportunity for intervention in close association with the gambling situation. This differs to some extent from previous studies; Neighbors and co-workers (21) studied a college student sample with primarily land-based gambling types, including playing cards for money, casino gambling, or lotteries. In the study by Hodgins and co-workers (23), an online intervention was delivered to a nontreatment-seeking sample among whom a majority reported gambling on electronic gambling machines. Theoretically, the strength of the intervention is to help individuals reflect on their level of gambling as perceived in comparison to the gambling of others and that the individuals may correct their misperceptions about how much others gamble (21). In this case, such a normative feedback intervention may be particularly helpful when provided directly by the gambling operator, thereby displaying—in close temporal association with the actual gambling situation—the true level of the individual's gambling on that operator's sites.

The present study has limitations: the first is that the results of the study cannot be compared to those of a formal control group, such as in a randomized controlled design, although non-completers were instead analyzed here as a control condition. Future studies should compare the present type of normative intervention to an “as-usual” condition or to another control group in order to better control for the possibility of the results being caused by regression toward the mean. It shall be borne in mind that despite the promising pattern seen after the normative intervention in the present study, it cannot be excluded that a decline in gambling, after a more intense gambling period, might be due to decreasing financial resources of the individuals, other barriers to gambling imposed by financial constraints or by families of the gambler, or other natural fluctuations in at-risk gamblers. For example, it has recently been shown that high-level gambling patterns may vary substantially over time (47), which is also in accordance with the fact that many individuals vary over time in their fulfillment of problem gambling definitions (13).

Moreover, the follow-up time in the study was limited. Several other intervention studies using real-world data on brief interventions carried out by the gambling operator have used follow-up periods that were as short, or shorter, compared to the present one. In contrast to the somewhat longer follow-up periods in studies carried out in other populations than in gambling operator clients (22), several studies using brief messages from a gambling operator have used short follow-up periods. These follow-up durations have included a number of days (16), or studied gambling outcomes in even closer proximity to the actual intervention (15). One study of an intervention of a loss-limit reminder from a Norwegian gambling operator included a 3-month follow-up, i.e., similar in duration to the secondary analysis of the present study (27). An exception is the 1-year follow-up of a telephone or letter intervention in Norway, which thereby, however, can be assumed to represent a more in-depth intervention than the brief and automated ones described in the present study and elsewhere (17).

Although a 4-week study period prior to the intervention was maintained, a secondary analysis was conducted, involving 12 weeks of follow-up post-intervention. The pre-intervention period in that analysis was maintained at 28 days, as the number of included individuals with a full study period would otherwise be more limited; individuals who entered the study during the month prior to the intervention would otherwise be missing in a longer pre-intervention period. Although the pre- and post-intervention periods do not have the same duration and therefore cannot be readily comparable, this analysis demonstrates no tendency for ADW values to return to pre-intervention levels. While this further supports the impression of a downward trend in wagering following the normative feedback intervention, the largest decrease may occur during the first weeks after the intervention. Further studies should address whether normative feedback interventions may need to be repeated in order to maintain a decrease in gambling over a longer follow-up period.

Gambling through other companies than the present one cannot be detected in the study. Likewise, as no actual self-report data were available, no diagnostic criteria were available, and therefore, beyond the sole reporting of the risk level from the test, no other measures of problem gambling or gambling disorder could be included. The aim of the study was to test a model for intervention by a gambling operator in individuals with a theoretical at-risk gambling pattern, or an own interest in taking a risk gambling test, rather than a clinical intervention in individuals presenting with a manifest gambling problem. Likewise, the present intervention included one specific gambling operator and was conducted in one specific geographical setting, where problem gambling is predominantly online-based (28). Therefore, the findings cannot readily be generalized to other gambling operators or to settings where a higher percentage of problem gamblers report land-based gambling.

In conclusion, a normative feedback intervention, asking questions about an individual's gambling patterns and her/his beliefs about peer gambling, may be associated with a decrease in gambling in online gamblers hypothesized to have a potentially hazardous gambling pattern. While such an intervention previously has not been studied in its direct administration from a gambling operator, it here proved at least feasible in the context of a state-owned gambling operator, although its further use in other types of gambling settings may need to be tested. The association between the normative intervention and the reduction in wagering was stronger in younger individuals and stronger in the short term in online casino gamblers. In a longer time frame, following up individuals for 12 weeks post-intervention, the association with reduced gambling patterns may be less pronounced. These results can be seen as promising, and although they should be interpreted with caution, they call for future studies in larger study samples and in other settings, including longer follow-up durations.

## Data Availability Statement

The datasets presented in this article are not readily available because a request for study data would be sent for review by the Ethics Committee and the company owning the data. Requests to access the datasets should be directed to anders_c.hakansson@med.lu.se.

## Ethics Statement

The studies involving human participants were reviewed and approved by Regionala etikprövningsnämnden Lund (Regional ethics authority Lund), Sweden. The patients/participants provided their written informed consent to participate in this study.

## Author Contributions

AH, AL, and KF were responsible of the overall project planning and data collection. Detailed study design was planned by AH, AL, KF, and JB. Statistical work was carried out by JB. JB and AH wrote the first drafts of the manuscript. TA, AL, and KF made substantial contributions to the text. Interpretation of data and results were made mainly by all authors. All authors approved the final paper and the submission.

## Funding

Overall research support from the Swedish state-owned gambling operator Svenska spel, including non-project-specific funding, and non-financial structural resource support to the present project.

## Conflict of Interest

AL and KF were employed by the state-owned Swedish gambling operator AB Svenska Spel, and work in the subdivision responsible of responsible gambling strategies, self-assessment tools etc. The study was carried out thanks to the overall support from this gambling operator. AH and his research group receive overall research funding from the same state-owned gambling operator. The company as such (except for the employees involved as co-authors) was not involved in the interpretation of data and the writing or editing of the paper. The remaining authors declare that the research was conducted in the absence of any commercial or financial relationships that could be construed as a potential conflict of interest.

## Publisher's Note

All claims expressed in this article are solely those of the authors and do not necessarily represent those of their affiliated organizations, or those of the publisher, the editors and the reviewers. Any product that may be evaluated in this article, or claim that may be made by its manufacturer, is not guaranteed or endorsed by the publisher.

## Supplementary Material

Table S1 Model selection for the structural submodel.

Table S2 Multivariate regression analyses.

## References

[1] American Psychiatric Association. Diagnostic and Statistical Manual of Psychiatric Disorders. Arlington, VA:American Psychiatric Publishing (2013).

[2] Calado F Griffiths M. Problem gambling worldwide: an update and systematic review of empirical research (2000-2015). J Behav Addict. (2016) 5:592–613. 10.1556/2006.5.2016.073 27784180 PMC5370365

[3] Williams RJ, Volberg, RA, Stevens, RMG,. The Population Prevalence of Problem Gambling: Methodological Influences, Standardized Rates, Jurisdictional Differences, Worldwide Trends. Report prepared for the Ontario Problem Gambling Research Centre the Ontario Ministry of Health Long Term Care. University of Lethbridge Research Repository (2012). Available online at: https://opus.uleth.ca/bitstream/handle/10133/3068/2012-PREVALENCE-OPGRC%20(2).pdf (accessed September 4, 2020).

[4] Potenza MN Balodis IM Derevensky J Grant JE Petry NM Verdejo-Garcia A. Gambling disorder. Nat Rev Dis Primers. (2019) 5:51. 10.1038/s41572-019-0099-7 31346179

[5] Oksanen A Savolainen L Sirola A Kaakinen M. Problem gambling and psychological distress: a cross-national perspective on the mediating effect of consumer debt and debt problems among emerging adults. Harm Red J. (2018) 15:45. 10.1186/s12954-018-0251-9 30176935 PMC6122437

[6] Håkansson A Widinghoff C. Indebtedness and problem gambling in a general population sample of online gamblers. Front Psychiatry. (2020) 11:7. 10.3389/fpsyt.2020.00007 32116832 PMC7016486

[7] Ronzitti S Soldini E Smith N Potenza MN Clerici M Bowden-Jones H. Current suicidal ideation in treatment-seeking individuals in the United Kingdom with gambling problems. Addict Behav. (2017) 74:33–40. 10.1016/j.addbeh.2017.05.032 28570912

[8] Karlsson A Håkansson A. Suicide, mortality and comorbidity in patients with pathological gambling – a nationwide register study. J Behav Addict. (2018) 7:1091–9. 10.1556/2006.7.2018.112 30427214 PMC6376387

[9] Gainsbury S Hing N Suhonen N. Professional help-seeking for gambling problems: awareness, barriers and motivators for treatment. J Gambl Stud. (2014) 30:503–19. 10.1007/s10899-013-9373-x 23494244

[10] Suurvali H Cordingley J Hodgins DC Cunningham J. Barriers to seeking help for gambling problems: a review of the empirical literature. J Gambl Stud. (2009) 25:407–24. 10.1007/s10899-009-9129-9 19551495

[11] Volberg R. Has there been a “feminization” of gambling and problem gambling in the United states? J Gambl Iss. (2003) 8. 10.4309/jgi.2003.8.7

[12] McCormack A Shorter GW Griffiths MD. An empirical study of gender differences in online gambling. J Gambl Stud. (2014) 30:71–88. 10.1007/s10899-012-9341-x 23097131

[13] Abbott MW Romild U Volberg RA. The prevalence, incidence, and gender and age-specific incidence of problem gambling: results of the Swedish longitudinal gambling study (Swelogs). Addiction. (2018) 113:699–707. 10.1111/add.14083 29105942

[14] Svensson J Romild U. Problem gambling features and gendered gambling domains amongst regular gamblers in a Swedish population-based study. Sex Roles. (2014) 70:240–54. 10.1007/s11199-014-0354-z 24634562 PMC3953604

[15] Auer MM Griffiths MD. Testing normative and self-appraisal feedback in an online slot-machine pop-up in a real-world setting. Front Psychol. (2015) 6:339. 10.3389/fpsyg.2015.00339 25852630 PMC4369874

[16] Auer MM Griffiths MD. Personalized behavioral feedback for online gamblers: a real world empirical study. Front Psychol. (2016) 7:1875. 10.3389/fpsyg.2016.01875 27965611 PMC5124696

[17] Jonsson J Hodgins DC Munck I Carlbring P. Reaching out to big losers leads to sustained reductions in gambling over 1 year: a randomized controlled trial of brief motivational contact. Addiction. (2020) 115:1522–31. 10.1111/add.14982 31977104

[18] Håkansson A Kostevski A Ekblad S. Gambling habits, gambling norms, and problem gambling in foreign born and native populations in Denmark – a general population survey. Addict Behav Rep. (2019) 9:100183. 10.1016/j.abrep.2019.100183 31193793 PMC6542756

[19] Larimer ME Neighbors C. Normative misperception and the impact of descriptive and injunctive norms on college student gambling. Psychol Addict Behav. (2003) 17:235–43. 10.1037/0893-164X.17.3.235 14498818

[20] Foster DW Neighbors C Rodriguez LM Lazorwitz B Gonzales R. Self-identification as a moderator of the relationship between gambling-related perceived norms and gambling behavior. J Gambl Stud. (2014) 30:125–40. 10.1007/s10899-012-9346-5 23143706 PMC4238910

[21] Neighbors C. Efficacy of personalized normative feedback as a brief intervention for college student gambling: a randomized controlled trial. J Consult Clin Psychol. (2015) 83:500–11. 10.1037/a0039125 26009785 PMC4939822

[22] Marchica L Derevensky J. Examining personalized feedback interventions for gambling disorders: a systematic review. J Behav Addict. (2016) 5:1–10. 10.1556/2006.5.2016.006 28092190 PMC5322985

[23] Hodgins DC Cunningham JA Murray R Hagopian S. Online self-directed interventions for gambling disorder: randomized controlled trial. J Gambl Stud. (2019) 35:635–51. 10.1007/s10899-019-09830-7 30701377

[24] van der Maas M Shi J Elton-Marshall T Hodgins DC Sanchez S Lobo DSS. Internet-based interventions for problem gambling: scoping review. JMIR Mental Health. (2019) 6:e65. 10.2196/mental.9419 30617046 PMC6329421

[25] Grande-Gosende A López-Núñez C García-Fernández G Derevensky J Fernández-Hermida JR. Systematic review of preventive programs for reducing problem gambling behaviors among young adults. J Gambl Stud. (2020) 36:1–22. 10.1007/s10899-019-09866-9 31168687

[26] Luquiens A Tanguy ML Lagadec M Benyamina A Aubin HJ Reynaud M. The efficacy of three modalities of internet-based psychotherapy for non-treatment-seeking online problem gamblers: a randomized controlled trial. J Med Internet Res. (2016) 18:e36. 10.2196/jmir.4752 26878894 PMC4771930

[27] Auer MM Hopfgartner N Griffiths MD. The effect of loss-limit reminders on gambling behavior: a real-world study of Norwegian gamblers. J Behav Addic. (2018) 7:1056–67. 10.1556/2006.7.2018.106 30418076 PMC6376395

[28] Håkansson A Mårdhed E Zaar M. Who seeks treatment when medicine opens the door to gambling disorder patients – psychiatric co-morbidity and heavy predominance of online gambling. Front Psychiatry. (2017) 8:255. 10.3389/fpsyt.2017.00255 29238309 PMC5712539

[29] The Swedish Government. En omreglerad spelmarknad. Betänkande av spellicensutredningen. A regulated gambling market. The gambling license bill. SOU 2017:30 (in Swedish, summary in English). The Swedish Government (2017). Available online at: https://www.regeringen.se/4969b7/contentassets/29291777554d47e49e717171e4eb5f83/en-omreglerad-spelmarknad-del-1-av-2-kapitel-1-21-sou-201730 (accessed September 4, 2020).

[30] Håkansson A Widinghoff C. Television gambling advertisements: extent and content of gambling advertisements with a focus on potential high-risk commercial messages. Addict Behav Rep. (2019) 9:100182. 10.1016/j.abrep.2019.100182 31193826 PMC6542735

[31] BBC. Sweden female gambling addicts outnumber men for first time. BBC (2019) Available online at: https://www.bbc.com/news/world-europe-47814630 (accessed September 4, 2020).

[32] Granero R Penelo E Stinchfield R Fernández-Aranda F Savvidou LG Fröberg F. del Pino-Gutiérrez A, Menchón JM, Jiménez-Murcia S. Is pathological gambling moderated by age? J Gambl Stud. (2014) 30:475–92. 10.1007/s10899-013-9369-6 23494243

[33] Petry NM. A comparison of young, middle-aged, and older adult treatment-seeking pathological gamblers. Gerontologist. (2002) 42:92–9. 10.1093/geront/42.1.92 11815703

[34] Griffiths MD Wood RT Parke J. Social responsibility tools in online gambling: a survey of attitudes and behavior among Internet gamblers. Cyberpsychol Behav. (2009) 12:413–26. 10.1089/cpb.2009.0062 19594379

[35] Forsström D Hesser H Carlbring P. Usage of a responsible gambling tool: a descriptive analysis and latent class analysis of user behavior. J Gambl Stud. (2016) 32:889–904. 10.1007/s10899-015-9590-6 26753878

[36] Auer M Griffiths MD. An Empirical Investigation of Theoretical Loss and Gambling Intensity. J Gambl Stud. (2014) 30:879–87. 10.1007/s10899-013-9376-7 23508851

[37] Neighbors C Lostutter TW Larimer MR Takushi RY. Measuring gambling outcomes among college students. J Gambl Stud. (2002) 18:339–60. 10.1023/A:1021013132430 12514914 PMC1797803

[38] R Core Team. R: A language and environment for statistical computing. R Foundation for Statistical Computing, Vienna, Austria. R Core Team (2019). Available online at: https://www.R-project.org/ (accessed March 18, 2022)

[39] Crisp BR Thomas SA Jackson AC Smith S Borrell J Ho WY. Not the same: a comparison of female and male clients seeking treatment from problem gambling counselling services. J Gambl Stud. (2004) 20:283–99. 10.1023/B:JOGS.0000040280.64348.d1 15353925

[40] Slutske WS Piasecki TM Deutsch AR Statham DJ Martin NG. Telescoping and gender differences in the time course of disordered gambling: evidence from a general population sample. Addiction. (2015) 110:144–51. 10.1111/add.12717 25171127 PMC4270904

[41] Blanco C Hasin DS Petry N Stinson FS Grant BF. Sex differences in subclinical and DSM-IV pathological gambling: results from the National Epidemiologic Survey on Alcohol and Related Conditions. Psychol Med. (2006) 36:943–53. 10.1017/S0033291706007410 16650342

[42] Ekholm O Eiberg S Davidsen M Holst M Larsen CV Juel K. The prevalence of problem gambling in Denmark in 2005 and 2010: a sociodemographic and socioeconomic characterization. J Gambl Stud. (2014) 30:1–10. 10.1007/s10899-012-9347-4 23138984

[43] Fröberg F Rosendahl IK Abbott M Romild U Tengström A Hallqvist J. The incidence of problem gambling in a representative cohort of Swedish female and male 16-24 year-olds by socio-demographic characteristics, in comparison with 25-44 year-olds. J Gambl Stud. (2015) 31:621–41. 10.1007/s10899-014-9450-9 24590609

[44] André F Claesdotter-Knutsson E Fridh M Delfin C Håkansson A Lindström M. A cross-sectional study on extensive gambling in adolescents. J Publ Health Res. (2021) 11:2498. 10.4081/jphr.2021.2498 34595902 PMC8874861

[45] Di Nicola M Ferri VR Moccia L Panaccione I Strangio AM Tedeschi D. Gender differences and psychopathological features associated with addictive behaviors in adolescents. Front Psychiatry. (2017) 8:256. 10.3389/fpsyt.2017.00256 29249992 PMC5716988

[46] Rodda SN Dowling NA Lubman DI. Gamblers seeking online help are active help-seekers: time to support autonomy and competence. Addict Behav. (2018) 87:272–5. 10.1016/j.addbeh.2018.06.001 29935737

[47] Muggleton N Parpart P Newall P Leake D Gathergood J Stewart N. The association between gambling and financial, social and health outcomes in big financial data. Nat Hum Behav. (2021) 5:319–26. 10.1038/s41562-020-01045-w 33542528

