# Supplementary material for: A Normative Feedback Intervention on Gambling Behavior—A Longitudinal Study of Post-Intervention Gambling Practices in At-Risk Gamblers
Source: Front Psychiatry. 2022 Mar 31;13:602846. doi: 10.3389/fpsyt.2022.602846 (PMC9008880; doi:10.3389/fpsyt.2022.602846)
Supplement: Table S1 — Model selection for the structural submodel. [file Table_1.docx]

| **Supplementary table s1. Model selection for the structural submodel** | | |  |
| --- | --- | --- | --- |
| *Variables included in the model* | *df* | *AIC* | *BIC* |
| Intervention mean change | 4 | 39532.52 | 39561.39 |
| Intervention slope change | 4 | 39457.06 | 39485.92 |
| Intervention mean change, intervention slope change | 5 | 39461.45 | 39497.54 |
| Period, intervention mean change | 5 | 39454.02 | 39490.10 |
| Period, intervention slope change | 5 | 39446.62 | 39482.70 |
| Period, intervention mean change, intervention slope change | 6 | 39451.71 | 39494.97 |
| First period, intervention mean change | 6 | 39507.84 | 39551.14 |
| First period, intervention slope change | 6 | 39432.12 | 39475.42 |
| First period, intervention mean change, intervention slope change | 7 | 39436.76 | 39487.28 |
| Period, first period, intervention mean change | 7 | 39433.16 | 39483.68 |
| Period, first period, intervention slope change | 7 | 39424.33 | 39474.84 |
| Period, first period, intervention mean change, intervention slope change | 8 | 39429.44 | 39487.17 |

| **Supplementary table s2. Multivariate regression analyses.** | | | |  | | |
| --- | --- | --- | --- | --- | --- | --- |
|  | Full model | | Sensitivity analysis 1 | | |  |
|  | *Estimate (95% CI)* | *p-value* | *Estimate (95% CI)* | | *p-value* |  |
| Period | -0.08 (-0.11, -0.04) | <.001 | -0.08 (-0.11, -0.05) | | <.001 |  |
| *First period* |  |  |  | |  |  |
| 1 (reference) | *1* |  |  | |  |  |
| 2 | -0.32 (-0.55, -0.09) | .006 |  | |  |  |
| 3 | -0.58 (-0.91, -0.26) | <.001 |  | |  |  |
| Intervention slope change | -0.29 (-0.39, -0.19) | <.001 | -0.20 (-0.30, -0.10) | | <.001 |  |
| Female sex | -0.08 (-0.39, 0.23) | .610 | -0.23 (-0.58, 0.11) | | .185 |  |
| Age (per 10 years) | 0.04 (-0.02, 0.09) | .163 | 0.04 (-0.01, 0.10) | | .131 |  |
| Entry method | -0.27 (-0.44, -0.09) | .002 | -0.22 (-0.40, -0.03) | | .022 |  |
| *Preferred gambling type* |  |  |  | |  |  |
| Betting (reference) | 1 |  | 1 | |  |  |
| Online casino | 0.41 (0.01, 0.81) | .046 | 0.44 (-0.01, 0.89) | | .057 |  |
| Other | -0.12 (-0.33, 0.08) | .237 | -0.10 (-0.31, 0.12) | | .379 |  |
| *Intervention feedback* |  |  |  | |  |  |
| Moderate estimation | 1 |  | 1 | |  |  |
| Over-estimation | 0.32 (0.10, 0.54) | .004 | 0.28 (0.06, 0.51) | | .015 |  |
| Non-completer | 0.15 (-0.07, 0.37) | .187 | 0.13 (-0.10, 0.37) | | .267 |  |
| Intervention x sex | -0.10 (-0.19, -0.02) | .021 | -0.05 (-0.14, 0.04) | | .304 |  |
| Intervention x age | 0.05 (0.04, 0.07) | <.001 | 0.04 (0.03, 0.06) | | <.001 |  |
| Intervention x entry | 0.00 (-0.04, 0.05) | .895 | 0.05 (0.01, 0.10) | | .028 |  |
| Intervention x casino | -0.15 (-0.26, -0.04) | .007 | -0.27 (-0.39, -0.16) | | <.001 |  |
| Intervention x other | 0.04 (-0.02, 0.09) | .169 | 0.00 (-0.05, 0.06) | | .862 |  |
| Intervention x over-estimation | -0.05 (-0.11, 0.01) | .120 | -0.04 (-0.10, 0.02) | | .187 |  |
| Intervention x non-completer | -0.02 (-0.08, 0.04) | .457 | -0.05 (-0.11, 0.01) | | .085 |  |

|  | Sensitivity analysis 2 | | Sensitivity analysis 3 | |
| --- | --- | --- | --- | --- |
|  | *Estimate (95% CI)* | *p-value* | *Estimate (95% CI)* | *p-value* |
| Period | 0.07 (0.05, 0.09) | <.001 | -0.02 (-0.04, 0.00) | .118 |
| *First period* |  |  |  |  |
| 1 (reference) | *1* |  | *1* |  |
| 2 | 0.20 (0.01, 0.38) | .040 | -0.10 (-0.27, 0.06) | .225 |
| 3 | -0.02 (-0.29, 0.24) | .866 | -0.29 (-0.53, -0.05) | .017 |
| Intervention slope change | -0.22 (-0.29, -0.15) | <.001 | -0.20 (-0.27, -0.14) | <.001 |
| Female sex | -0.05 (-0.30, 0.19) | .679 | -0.04 (-0.27, 0.19) | .725 |
| Age (per 10 years) | 0.00 (-0.04, 0.04) | .944 | 0.02 (-0.02, 0.06) | .388 |
| Entry method | -0.30 (-0.43, -0.16) | <.001 | -0.20 (-0.32, -0.07) | .002 |
| *Preferred gambling type* |  |  |  |  |
| Betting (reference) | 1 |  | 1 |  |
| Online casino | 0.73 (0.41, 1.05) | <.001 | 0.46 (0.17, 0.75) | .002 |
| Other | -0.27 (-0.43, -0.11) | .001 | -0.17 (-0.32, -0.02) | .025 |
| *Intervention feedback* |  |  |  |  |
| Moderate estimation |  |  | 1 |  |
| Over-estimation | 0.35 (0.18, 0.52) | <.001 | 0.28 (0.13, 0.44) | <.001 |
| Non-completer | 0.19 (0.01, 0.36) | .037 | 0.13 (-0.03, 0.29) | .108 |
| Intervention x sex | -0.01 (-0.08, 0.05) | .655 | -0.06 (-0.11, 0.00) | .054 |
| Intervention x age | 0.02 (0.00, 0.03) | .005 | 0.03 (0.02, 0.04) | <.001 |
| Intervention x entry | 0.01 (-0.03, 0.04) | .731 | 0.00 (-0.03, 0.03) | .883 |
| Intervention x casino | -0.06 (-0.15, 0.02) | .142 | -0.10 (-0.17, -0.03) | .008 |
| Intervention x other | 0.02 (-0.02, 0.06) | .377 | 0.03 (-0.01, 0.06) | .186 |
| Intervention x over-estimation | -0.03 (-0.07, 0.01) | .187 | -0.04 (-0.08, 0.00) | .032 |
| Intervention x non-completer | -0.02 (-0.07, 0.02) | .318 | -0.03 (-0.07, 0.02) | .226 |
